# Cervical spine myxoid liposarcoma in a 30-year-old male: a rare case report

**DOI:** 10.1097/RC9.0000000000000047

**Published:** 2026-05-19

**Authors:** Ibrahim Fathallah, Ahmed Al-Talep, Abd Alrhman Alajrd, Omar Alsabbagh, Ahmad Alhamoud

**Affiliations:** a Faculty of Medicine, Homs University, Homs, Syria; b Faculty of Medicine, Hama University, Hama, Syria; c Department of Neurosurgery, Damascus Hospital, Damascus, Syria; d Faculty of Medicine, Al-Fourat University, Deir ez-Zor, Syria

**Keywords:** cervical spine, liposarcoma, myxoid liposarcoma

## Abstract

**Introduction and clinical importance:**

Myxoid liposarcoma (MLS) is a rare soft tissue sarcoma subtype, typically affecting adults. Spinal involvement is exceptionally uncommon, requiring careful diagnosis and management. We report a rare primary cervical spine case.

**Case presentation:**

A 30-year-old man came to the neurology clinic transferred from a general surgeon with weakness and stiffness in both legs after a biopsy of a deep neck mass. He came to the general surgeon with a history of neck swelling with mild dysphagia. Magnetic resonance imaging (MRI) showed a large mass from C5 to T3 with spinal curvature. The surgeons removed the tumor, which turned out to be primary MLS. The patient regained full leg movement after physiotherapy and is currently receiving adjuvant therapy.

**Clinical discussion:**

MLS develops from primitive mesenchymal cells rather than mature fat and can spread to unusual sites like the spine. MRI is valuable for diagnosis, and complete surgical removal is the main treatment, with radiotherapy or chemotherapy considered only when necessary.

**Conclusion:**

This case emphasizes considering MLS in deep cervical masses and the importance of early treatment significantly contributed to the patient’s full recovery.

## Introduction

Liposarcoma is the most occurring soft tissue sarcoma (STS) among adults. There are five histological categories: well-differentiated, dedifferentiated, myxoid, round cell, and pleomorphic^[^1^]^. Myxoid liposarcoma (MLS) is known as the second most common histological type, and most cases are between 40 and 60 years old^[^2^]^. MLS is a disease seen in young adults, with the same occurring rate in both males and females^[^3^]^. Liposarcoma comprises 5% of all STSs, and 15%–20% of liposarcomas are seen in adults, commonly found in the buttocks, retroperitoneum, trunk, ankle, and proximal limb girdle^[^1^]^. MLS represents about 30% of all liposarcoma cases^[^4^]^. The most frequent anatomical site for MLS is the lower extremities^[^4^]^. Despite liposarcoma being the most common STS in adults, its occurrence in the head and neck region is rare, constituting less than 5% of all cases^[^5^]^. Rarely, primary or secondary spinal involvement transpires, with few case reports documented^[^2^]^. Symptoms often develop over time as a painless mass in deep soft tissue^[^3^]^. The unique clinical features of MLS may require specialized diagnostic and treatment protocols, as it is prone to spinal metastasis, differentiating it from most STSs^[^6^]^. This study presents an exceptionally rare case of primary MLS arising in the cervical spine, an anatomical location that is highly unusual for this malignancy. This work is also reported in line with SCARE criteria, thereby increasing the report’s transparency and quality^[^7^]^.

**HIGHLIGHTS**

- It is very rare for myxoid liposarcoma (MLS) to appear in the cervical spine; only a handful of cases have been described.
- MLS typically develops deep in the soft tissues and may not cause pain at first, but it may lead to leg weakness or paralysis.
- Magnetic resonance imaging and a biopsy are crucial to make the correct diagnosis, and surgery to remove the tumor along with stabilizing the spine usually works well.
- When surgery is done promptly and followed by proper rehabilitation, patients can recover their neurological function completely, even in large cervical MLS.

## Case presentation

A 30-year-old male patient was referred by a general surgeon with a history of paraparesis following a biopsy of a cervical mass. Upon presentation, the patient was conscious and cooperative, with spastic paralysis affecting both lower limbs. The patient neglected the complaint after 3 weeks and was transferred to the neurosurgical clinic for the most recent complaint. Upon questioning, it was revealed that there was no prior surgical or medication history and no prior medical history. Clinical examination did not reveal any palpable swelling in the neck, as the tumor was situated deeply. Neurological assessment demonstrated spastic paraparesis of the lower extremities, graded as 1/5, accompanied by hyperreflexia and clonus. Regarding the ASIA scale L.L motor dysfunction 0, L.L sensory deficit 2, trunk, sensory deficit 2, sphincter dysfunction 0, total 4 blood tests were within normal limits. Urgent magnetic resonance imaging (MRI) with contrast administration revealed a large, ill-defined, and heterogeneous mass in the soft tissues surrounding the cervical and upper thoracic vertebrae, extending from C5 to T3, with the development of kyphotic deformity. The lesion measured over 7 × 8.5 × 12 cm encasing both vertebral arteries and extended to the spinal canal through both neural foramina of C6 and C7, also to the space retro to the posterior elements.

**Figure 1.**
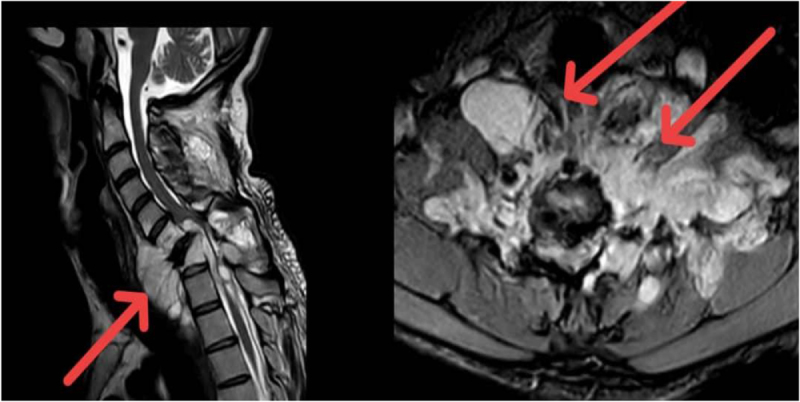
Sagittal T2 view shows the lesion (left) and the pathogenic fracture of the vertebral bodies and the invasion of the canal with anterior kyphosis. Axial T2 view shows the lesion in the neck and extended to the canal (right).

**Figure 2.**
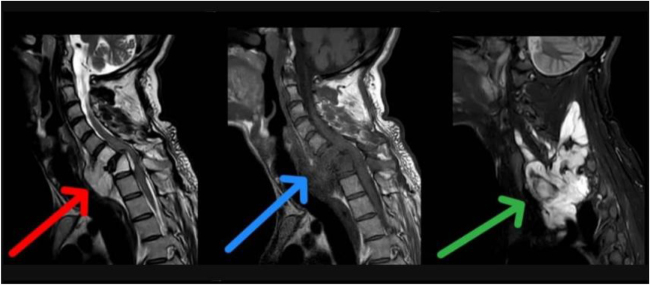
Sagittal MRI scan shows the lesion. T2 (red) T1 (blue) STIR (green).

**Figure 3.**
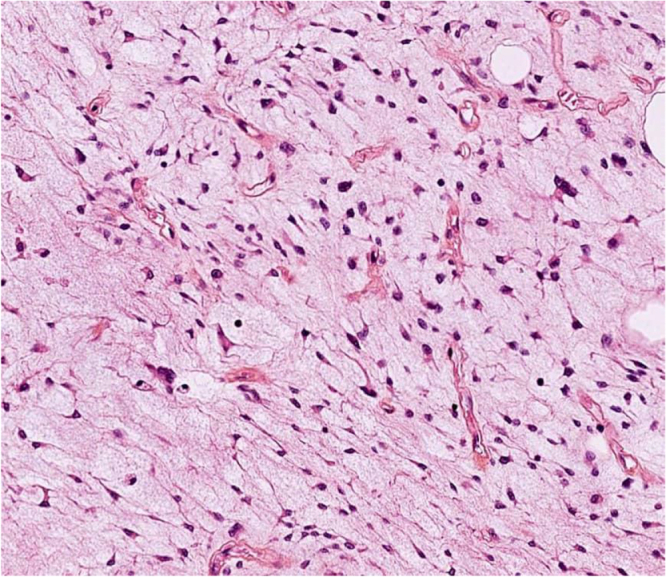
Shows fibroadipose and skeletal muscle tissue with an adipose tissue tumor composed of lobular architecture with low cellularity in the center and increased cellularity in the periphery of lobules. The tumor cells are composed of atypical lipoblast cells with eccentric pleomorphic nuclei and fat vacuoles with foci of high-grade cells. The center of lobules shows myxoid material. The tumor exhibits infiltration of the skeletal muscle and bone tissue. The backgrounds show necrosis, fibrosis, and foci of chronic inflammation.

**Figure 4.**
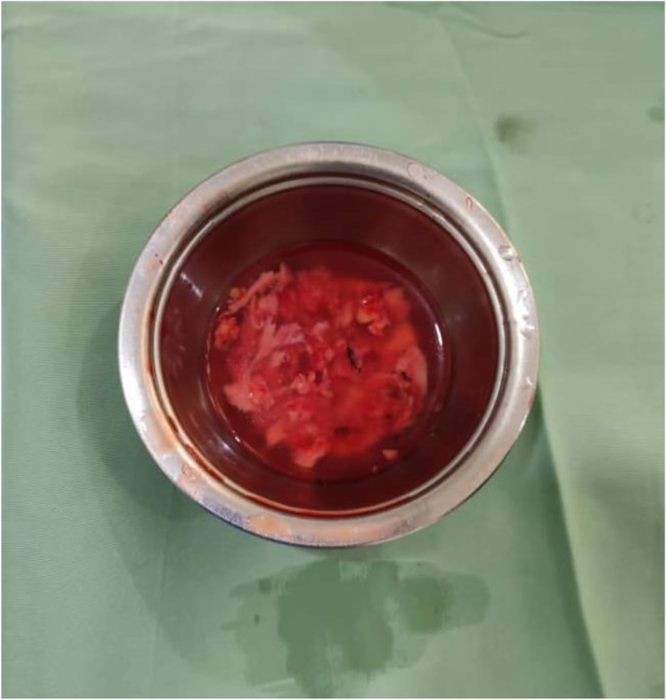
The tumor after removal.

**Figure 5.**
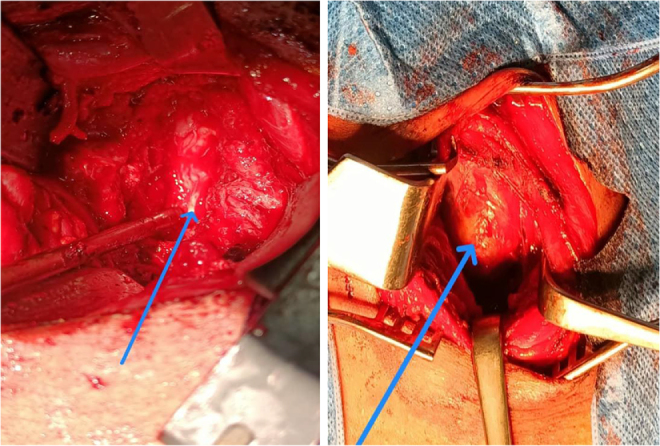
Intraoperative photo shows the spinal cord post-resection of the tumor and post-corpectomy (left). The neck mass intraoperative photo before resection (right).

**Figure 6.**
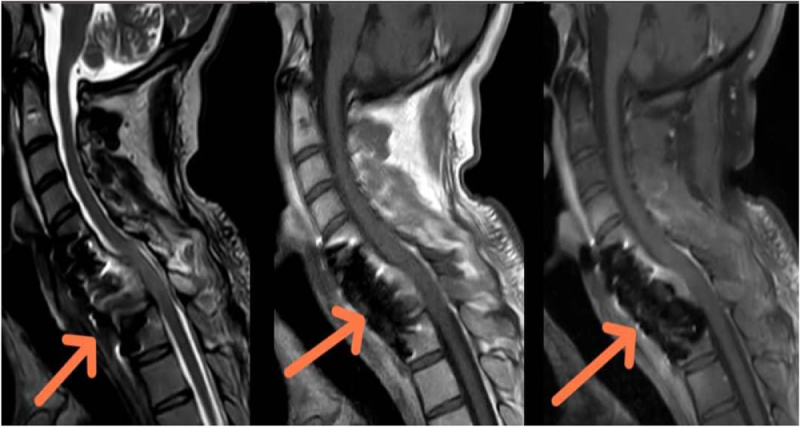
Postoperative MRI shows the artifact from the fixation device.

**Figure 7.**
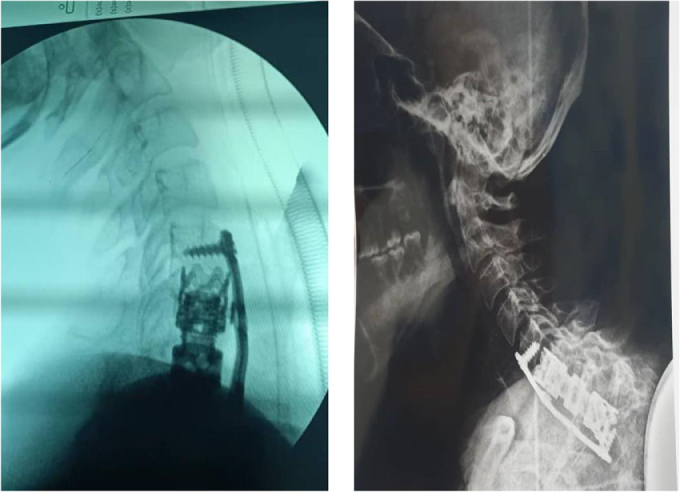
Intraoperative fluoroscopy shows the good alignment of the fixation device (left). Postoperative X-ray (right).

Edematous changes in C6 and C7 vertebral bodies associated with the compression fracture of C7 vertebra with retropulsion of vertebral body into spinal canal, causing 40% estimated stenosis of thecal sac and spinal cord signal changes, with the development of kyphotic deformity. The lesion appears hyperintense on T2 and hypointense on T1, with marked heterogeneous enhancement in post-contrast images (Figs 1 and 2). These findings raised suspicion for malignancy. Histopathological examination of the excised specimen revealed a soft, pale gray fibrofatty tissue. The lesion was encapsulated and contained a small amount of pus. The final diagnosis was confirmed as MLS (Fig. 3). Regarding the classification, it is resection status unknown due to the large tumor size and the necessity of performing the operation in two stages. The patient was prepared for urgent surgical intervention, linear incision parallel to the median border of the right sternocleidomastoid muscle medial to the carotid artery approach. During the approach, the tumor, which was strongly adherent to the vertebral bodies, was excised (Figs 4 and 5). Subsequently, a C6–C7 corpectomy and fusion were performed using an expandable cage and plate (Figs 6 and 7). Cervical spine surgery instruments were used (clawed _ Caspar retractors _ kerrison rongeurs_ curettes _maxillary forceps) Bipolar and monopolar. The operation took about 6 hours, and there was no more bleeding about 100cc. During the approach, I used nasogastric tube to help me in isolating the esophagus. Regarding the spinal alignment, the patient exhibits good and stable cervical motion post-surgery, along with spinal cord decompression and full restoration of movement. Postoperatively, the patient received ceftriaxone 1 gram twice daily, pantoprazole 40 milligrams once daily, and dexamethasone 4 milligrams once daily for 5 days, along with injectable tramadol 50 milligrams twice daily for 3 days, followed by as-needed administration. The patient was admitted to the intensive care unit for 2 days of monitoring. Chest and limb physiotherapy was initiated, followed by a gradual introduction of clear fluids, soft foods, and then solid foods. Regarding the adjuvant treatment, the patient was residing in a remote area with limited capacity and resources, and was thus referred to an oncology center to receive the adjuvant therapy. The patient is currently undergoing treatment. The patient was discharged without complications and commenced rehabilitation, achieving a good and complete recovery of motor function.

## Discussion

Primitive mesenchymal cells, not mature adipose tissue, are usually the source of liposarcoma^[^8,9^]^. Typically, MLSs are found on the limbs, especially the thighs. Due to the abundance of fat tissue at these locations, MLSs are more inclined to spread to extra-pulmonary sites like soft tissue, retroperitoneum, mediastinum, chest wall, peritoneal surface, and heart than other types of liposarcomas, which usually distributed to the lungs^[^1,10^]^. According to recent reports, the most frequent location of metastasis in MLS is the skeleton^[^10,11^]^. Compared to multiple myeloma, lymphoma, and metastatic disease, primary spine tumors are more uncommon lesions^[^12^]^.Even for metastases, the cervical spine is a rare site^[^13,14^]^.The primary site of the lesion in this case was the C5, C6, and C7 vertebrae. There was no evidence to suggest that the tumor was a metastasis from a primary tumor located in another part of the body. This finding confirms that the tumor is primary rather than secondary in origin. One of the most helpful methods to assess the anatomy of spinal tumors is MRI^[^12,15^]^. According to Noble *et al*, MRI is the most sensitive technique for detecting bone metastases in MLS, and negative bone scan results do not rule out bone metastases^[^16^]^. Imaging techniques such as ultrasound, computed tomography (CT), and MRI play a crucial role in assessing the lesion’s size, location, adipocytic composition, involvement of adjacent tissues, and its relationship with neurovascular structures, as well as in identifying possible metastases^[^17,18^]^. Key radiologic features that help differentiate liposarcoma from lipoma include the presence of septa thicker than 2 mm, nodular or globular non-adipose components within or adjacent to the lesion, and non-adipose tissue making up more than 25% of the total mass^[^18,19^]^. These findings support the consideration of liposarcoma when CT or MRI reveals proliferative fatty lesions containing myxoid components and enhanced vascularity, which are atypical for benign lipomas^[^20^]^. CT has a limited part in STS imaging. MRI is superior to CT for locoregional staging as it provides far superior soft towel resolution, and in addition MRI can produce images in any aeroplane or multiple aeroplanes to more optimally match familiar anatomical, surgical, or indeed conventional radiographic exposures. Certain features on CT are considered labels for aggressive features, including periphery irregularity, infiltration into conterminous organs, calcification, necrosis, and hypervascularity. For assessment of osseous involvement, which includes calcification, cortical destruction, and endosteal and periosteal response, MRI is either no better than or inferior to CT or indeed plain film radiography^[^21^]^. Despite the tumor’s proximity to critical structures in the head and neck, complete surgical excision remains the primary treatment of choice. Often, the main tumor is accompanied by satellite nodules, making comprehensive resection essential to minimize the risk of recurrence due to incomplete removal. Nevertheless, metastasis is uncommon in low-grade liposarcomas, such as well-differentiated and myxoid subtypes^[^19,22^]^. Radiotherapy is generally not the first-line treatment, but it has been used postoperatively, especially in MLSs that are more radiosensitive or in cases where surgical removal is not feasible. Postoperative radiotherapy can play an important role in reducing the risk of local recurrence, particularly in high-grade tumors, those without clear resection margins, large lesions, tumors showing aggressive biological behavior, or those situated in anatomically challenging locations. Interestingly, two reported cases of liposarcoma were managed with carbon ion radiotherapy^[^22^]^. Chemotherapy is most often considered in the setting of metastatic disease and has, in some instances, been combined with radiotherapy. Nevertheless, its therapeutic value in liposarcoma remains controversial and continues to be a subject of investigation^[^23,24^]^.

Regarding treatment, complete resection is the preferred approach. Surgical resection and radiation therapy are usually sufficient for local control^[^1^]^. In the present case, complete surgical resection of the tumor was performed and was thus referred to an oncology center to receive the adjuvant therapy. The patient is currently undergoing treatment.

## Conclusion

MLS is a rare tumor and is especially uncommon in the cervical spine. Careful evaluation with MRI and histopathological examination is crucial for an accurate diagnosis. Complete surgical removal provides the best chance for recovery, while radiation or chemotherapy is generally reserved for cases that are advanced or cannot be fully removed. This case underlines the need to consider primary MLS in the differential diagnosis of deep cervical masses and the importance of early treatment to achieve the best outcomes.

## Data Availability

Data sharing is not applicable to this article as no datasets were generated or analyzed during the current study.
